# The association between air pollution and obesity: an umbrella review of meta-analyses and systematic reviews

**DOI:** 10.1186/s12889-024-19370-4

**Published:** 2024-07-11

**Authors:** Chengwen Luo, Ting Wei, Weicong Jiang, Yu-pei Yang, Mei-Xian Zhang, Cai-Lian Xiong, Tao-Hsin Tung

**Affiliations:** 1 Evidence-based Medicine Center, Taizhou Hospital of Zhejiang Province affiliated to Wenzhou Medical University, Linhai, Zhejiang China; 2 Department of Bioinformatics and Biostatistics, School of Life Sciences and Biotechnology, Shanghai Jiao Tong University, Shanghai, China; 3 Department of Financial Markets, Linhai Rural Commercial Bank, Linhai, China; 4 Department of Hematology, Taizhou Hospital of Zhejiang Province affiliated to Wenzhou Medical University, Linhai, China; 5 Department of Nosocomial Infection Control, Taizhou Hospital of Zhejiang Province affiliated to Wenzhou Medical University, Linhai, China; 6 Taizhou Institute of Medicine, Health and New Drug Clinical Research, Taizhou, China

**Keywords:** Air pollution, Obesity, Umbrella review, Meta-analysis, AMSTAR 2

## Abstract

The objective of this umbrella review was to investigate comprehensive and synthesized evidence of the association between ambient air pollution and obesity based on the current systematic reviews and meta-analyses. Related studies from databases including PubMed, EMBASE, Web of Science, and the Cochrane Library, published before July 16, 2023, were considered in the analysis. All selected systematic reviews and meta-analyses were included in accordance with PRISMA guidelines. The risk of bias and the methodological quality were evaluated using the AMSTAR 2 tool. The protocol for this umbrella review was documented in PROSPERO with the registration number: CRD42023450191. This umbrella review identified 7 studies, including 5 meta-analyses and 2 systematic reviews, to assess the impacts of air pollutants on obesity. Commonly examined air pollutants included PM_1_, PM_2.5_, PM_10_, NO_2_, SO_2_, O_3_. Most of the included studies presented that air pollution exposure was positively associated with the increased risk of obesity. The impact of air pollution on obesity varied by different ambient air pollutants. This study provided compelling evidence that exposure to air pollution had a positive association with the risk of obesity. These findings further indicate the importance of strengthening air pollution prevention and control. Future studies should elucidate the possible mechanisms and pathways linking air pollution to obesity.

**Supplementary Information:**

The online version contains supplementary material available at 10.1186/s12889-024-19370-4.

## Introduction

Obesity is a chronic and complex physical and mental disease, which is increasingly prevalent in the world. The number of obese people worldwide has nearly tripled since 1975 [1]. It can be seen that the global population is moving towards overall obesity at a rapid rate. Data from the World Health Organization (WHO) indicated that in 2016, there were nearly 2 billion overweight adults around the world, with more than 650 million of them obese [2]. Obesity is a chronic metabolic disease caused by many factors, which is characterized by the disorder of energy metabolism and excessive accumulation of fat. Therefore, obesity will not only bring a series of harm to the weight itself and metabolism, but also cause many diseases and cause a huge burden of disease. The obesity epidemic is a serious threat to human health. The Global Burden of Obesity Study shows that the number of deaths due to Body Mass Index (BMI) more than doubled globally from 1990 to 2017 [3].

There is growing evidence that air pollution is one of the important risk factors for obesity [4, 5]. Air pollutants mainly include particulate matter (PM_1_, PM_2.5_, PM_10_, etc.), O_3_, CO, NO_2_, sulfide, etc., and their complex mixtures. The rapid expansion of industrialization and fossil fuel economy have coincided with unprecedented levels of global air pollution, due to the lack of effective measures for environmental protection [6]. The environmental pollutant PM_2.5_ was the fifth leading risk factor for death in 2015, with 4.2 million deaths worldwide due to PM_2.5_ exposure, accounting for 7.6% of all global deaths that year [7]. Many studies have reported that exposure to air pollution was associated with many adverse health events [8, 9]. For example, the study conducted in the US, followed by 3.9 million military veterans from 2010 to 2018, indicated that the increase in annual mean PM_2.5_ concentration significantly increased the risk of obesity [10]. In a study of 68,000 subjects aged 30–79 years in five provinces in southwest China, researchers reported that PM_1_, PM_2.5_, and PM_10_ exposures were positively correlated with obesity [11]. Animal studies have also indicated that particulate matter exposure could activate genes related to fat production in adipose tissue, leading to increased fat cell size, increased fat volume and visceral fat volume, and even weight gain [12, 13].

There have been numerous epidemiologic studies to assess the relationship between ambient air pollution and overweight/ obesity/ weight status [14–19]. However, the effects of air pollution on obesity appear to be very sophisticated and vary by population, pollutant type, and pollution severity [20]. Additionally, growing systematic reviews and meta-analyses have also addressed the effects of air pollutants on the risk of obesity [21–23]. Nevertheless, the results of some meta-analyses were different. For example, a meta-analysis focused on adult obesity indicated that the risk of obesity was not associated with exposure to PM_2.5_ [21]. In contrast to this finding, another meta-analysis showed a significant positive association between PM_2.5_ exposure and obesity [22]. Therefore, this study aimed to carry out an umbrella review to summarize the association between air pollution and obesity. This umbrella review would provide a synthesized comprehension of the influence of air pollution on obesity and give useful information for further research.

## Methods

### Search strategy

Studies for this umbrella review were accessed through searches of EMBASE, PubMed, the Cochrane Library, and Web of Science for relevant references without language limitations, from onset to July 16, 2023 (Supplementary Table 1). The search string used “air quality”, “air pollutant*”, “air pollution”, “fine particle”, “particulate matter”, “ozone”, “nitrogen oxide”, “sulfur dioxide”, “carbon monoxide”, “body mass index”, “BMI”, “overweight”, “obese”, “obesity”, “adiposity”, “weight”, “body fat”, “waist-to-hip”, “waist circumference”, “waist-to-height”, “visceral fat index”, “fat mass”, “fat-free mass”, “meta-analys*”, and “systematic review*”. The following search string “(air quality OR air pollution OR air pollutant* OR fine particle OR particulate matter OR carbon monoxide OR sulfur dioxide OR nitrogen oxide OR ozone) AND (body mass index OR obese OR obesity OR overweight OR adiposity OR BMI OR weight OR body fat OR waist-to-height OR waist-to-hip OR waist circumference OR visceral fat index OR fat mass OR fat-free mass) AND (Systematic review* OR meta-analys*)” was used to search for the possible articles. There were no restrictions on language. The whole search strategy met the requirements of the Preferred Reporting Project for Systematic Review and Meta-Analysis 2020 (PRISMA 2020) guidelines [24]. The protocol for this umbrella review was documented in PROSPERO with the registration number: CRD42023450191.

### Study selection

There were several inclusion criteria in this study as follows: (1) systemic review or meta-analysis; (2) the exposure of the study should relate to air pollution; and (3) the outcome should concentrate on obesity. Two authors (Chengwen Luo and Ting Wei) independently reviewed the titles, abstracts, and full text to identify the relevant research; any discrepancies were settled via discussion with a third researcher (Tao-Hsin Tung).

### Data extraction

We reviewed the systematic and meta-analysis papers, and explored the relationship between air pollution and obesity. Information was collected from the selected studies, including first author, published year, number of researches included, study purpose, air pollutants, outcome, effect size (risk ratio [RR], odds ratio [OR], or β), 95% confidence interval (95% CI), p-value, number of cases, heterogeneity degree I^2^, as well as study quality.

### Study quality assessment

The AMSTAR 2 (A MeaSurement Tool to Assess systematic Reviews) guidelines were used to assess the methodological quality of each included study. The AMSTAR 2 consists of 16 items that systematically score evidence-based medicine studies [25, 26]. AMSTAR 2 did not provide an overall score, as a high score may overlook some serious methodological deficiencies [27]. Hence, this quality rating tool is considered a reliable and valid method for evaluating the quality of the systematic review/ meta-analysis that involves both observational and interventional research [28]. Different from tools such as the risk of bias commonly used in non-randomized intervention studies, AMSTAR 2 assessed the process of selecting a study design for inclusion, the reason for excluding studies, the source of primary research funding, and any conflicts of interest among reviewers [29]. AMSTAR-2 is commonly used for the umbrella review [30–32].

### Epidemiological credibility assessment

High epidemiological credibility denotes the strongest evidence, with no signs of significant variance or bias [33]. The relationships included in this study are classified into the following types [34]. First, a “persuasive” relationship should meet the following criteria: statistical significance p-value < 0.000001 for the random effects model, number of cases greater than 1000, low degree of heterogeneity of included studies (I2 < 50%), 95% CI(excluding zero values), and no evidence of small study effects and significant bias. Second, “highly recommended” association includes statistical significance of a p-value < 0.000001, number of cases greater than 1000, and most studies reporting a significant effect. Third, the “recommended” association was supported by more than 1,000 cases, with a significant effect (p-value < 0.001). Fourth, a nominally significant association (p-value < 0.05) was considered “weak” evidence.

## Results

### Literature search

**Fig. 1:**
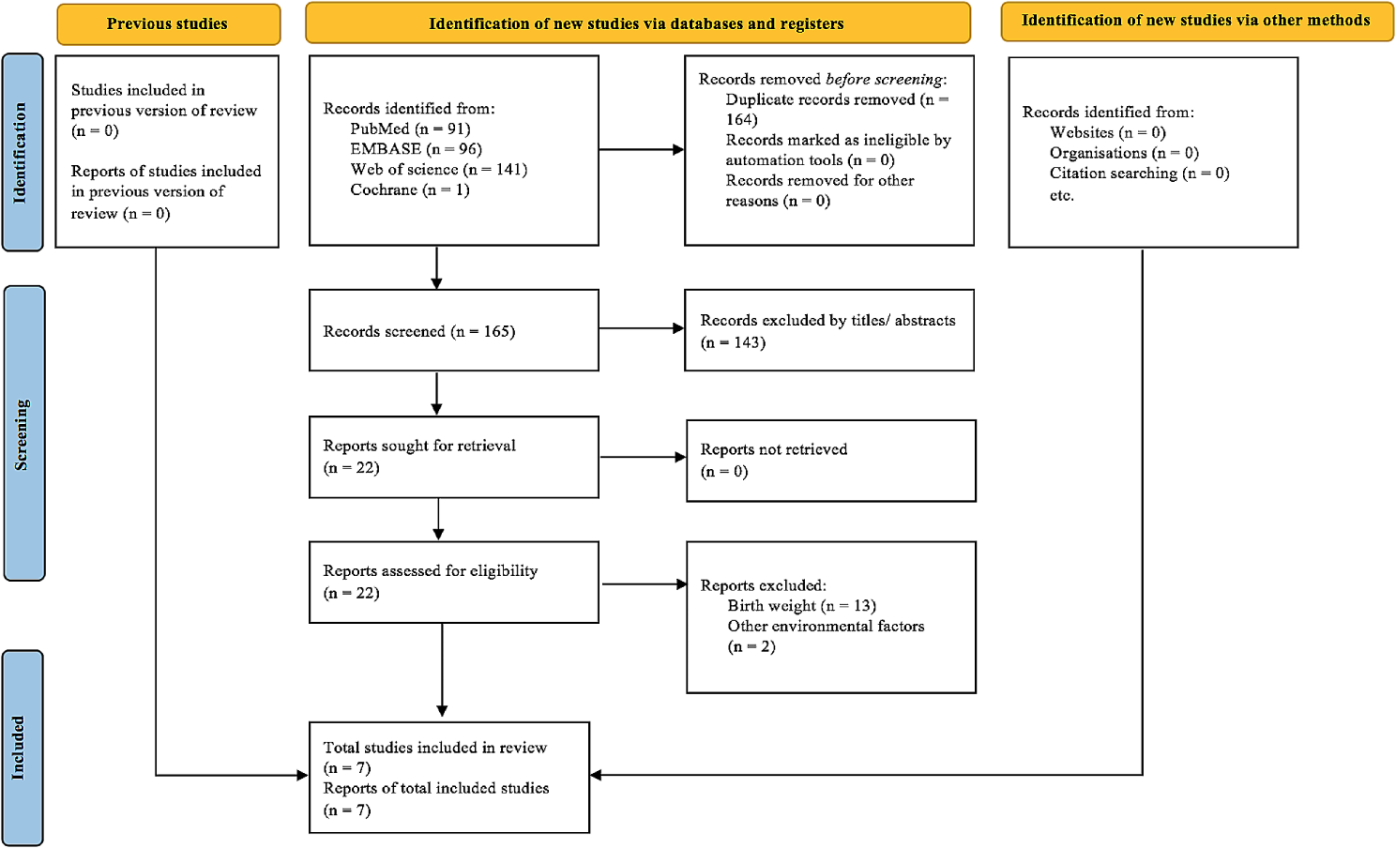
PRISMA flow chart

After carefully searching the electronic databases, 329 articles were obtained (96 from the EMBASE, 1 from the Cochrane Library, 141 from the Web of Science, and 91 from the PubMed) (Fig. 1). Considering the lack of a peer-review process, studies from preprint platforms were not included. Based on PRISMA, we finally included 7 articles (5 with and 2 without meta-analyses) [20–23, 35–37].

### Characteristics of the studies

The summarized characteristics of the included studies were listed in Table 1. All studies were published from 2018 to 2022. The most prevalent forms of research on outcomes are obesity, overweight, and BMI. Reviews related to obesity have focused on the following air pollutants: particular matter (PM_1_, PM_2.5_, and PM_10_), nitrogen oxide (NO_2_), sulfur dioxide (SO_2_), and ozone (O_3_). Each meta-analysis/ systematic review included 5 to 26 primary studies. The incremental levels of air pollutants reported in the included reviews varied, with some directly using the incremental levels of air pollutants in the primary study and others converting to a uniform incremental level (10 µg/m^3^).

**Table 1:** Characteristics of included studies

No.	Citation (First author et al., year)	Types	Number of studies	Purpose	Air pollutants	Outcome Effect on obesity/ BMI OR or RR (95% CI)/ β (95% CI)	*p*-value	Number of cases	I^2^	Study quality (AMSTAR 2) rating
1	Parasin, N et al., 2021	Meta-analysis	8	To assess the incidence of obesity in children due to air pollution using a systematic review and meta-analysis.	PM_2.5_ PM_10_ NO_2_	1.06 (1.02, 1.10)/ – 1.07 (1.04, 1.10) / – 1.10 (1.04, 1.16) / –	< 0.001/ – < 0.001/ – < 0.001/ –	> 1000/ – > 1000/ – > 1000/ –	44%/ – 73%/ – 55%/ –	Low
2	Huang, C et al., 2022	Meta-analysis	15	To assess the relationship between childhood exposure to air pollutants with obesity and weight status among children and adolescents.	PM_1_ PM_2.5_ PM_10_ NO_2_ O_3_	1.41 (1.30, 1.53) / – 1.28 (1.13, 1.45)/ 0.11(0.05, 0.17) 1.12 (1.06, 1.18)/ 0.08 (0.03, 0.12) 1.11 (1.06, 1.18)/ 0.03 (0.01, 0.04) 1.08 (0.99, 1.18) / –	< 0.05/ – < 0.05/ < 0.05 < 0.05/ < 0.05 < 0.05/ < 0.05 > 0.05/ –	> 1000/ – > 1000/ > 1000 > 1000/ > 1000 > 1000/ > 1000 > 1000/ –	0.0%/ – 86.3%/ 82.6% 85.9%/ 89.1% 84.1%/ 48.6% 71.5%/ –	Low
3	Bahreynian, M et al., 2020	Meta-analysis	5	To overview the human studies on the association of exposure to ambient Particulate Matter (PM) with childhood obesity.	PM_2.5_ PM_10_	–/ 0.035 (-0.099, 0.169) –/ 0.034 (0.007, 0.061)	–/ 0.606 –/ 0.015	–/ > 1000 –/ > 1000	–/ 95.30% –/ 16.60%	Low
4	Huang, S et al., 2020	Meta-analysis	10	To evaluate the effect of long-term exposure to ambient air pollutants on body weight status in adults.	PM_2.5_ PM_10_ NO_2_ SO_2_ O_3_	1.21 (0.94, 1.56)/ 0.34 (0.30, 0.38) 1.06 (0.98, 1.14)/ 0.03 (-0.26, 0.32) 1.13 (1.01, 1.26)/ 0.24 (-0.18, 0.67) 1.04 (1.01, 1.06)/ – 1.07 (1.02, 1.13)/ 0.21 (0.17, 0.24)	0.145/ < 0.001 0.137/ 0.815 0.034/ 0.261 0.003/ – 0.010/ < 0.001	> 1000/ > 1000 > 1000/ > 1000 > 1000/ > 1000 > 1000/ > 1000 > 1000/ > 1000	97.5%/ 0.0% 94.0%/ 94.4% 94.4%/ 98.6% 6.4%/ – 95.2%/ 0.0%	Low
5	Lin, L et al., 2022	Meta-analysis	26	To explore and study the role of PM on obesity.	PM_2.5_ PM_10_	1.159 (1.111, 1.209)/ – 1.092 (1.070, 1.116)/ –	< 0.001/ – < 0.001/ –	> 1000/ – > 1000/ –	99.2%/ – 97.8%/ –	Low
6	An, R et al., 2018	Systematic review	16	To systematically review scientific evidence regarding the influence of ambient air pollution on body weight status.	PM NO_2_ SO_2_ O_3_	Among 66 reported associations between air pollution and body weight status, 29 (44%) found air positive association, 29 (44%) reported a null finding, and 8 (12%) found negative association.	–	–	–	Low
7	LaKind, JS et al., 2021	Systematic review	8	To evaluate associations between ozone and effects on weight.	O_3_	The results did not indicate a consistent association between O_3_ exposure and effects on weight.	–	–	–	Low

Among the seven included studies, three reported on the impact of air pollutants on obesity among children and adolescents [23, 35, 36]. One study focused on the relationship between air pollutants and obesity among adults [21]. The other three studies involved both adults and children [20, 22, 37]. All seven included meta-analyses/ systematic reviews were of “low” quality based on the AMSTAR 2 criteria. In addition, according to the classification of epidemiological credibility, only two meta-analyses could be considered as the “recommended” epidemiological credibility of the association between air pollution and obesity [22, 35]. Another three meta-analyses were classified as having ‘weak evidence’ [21, 23, 36].

### Associations between air pollution and obesity

Amongst the two included systematic reviews, An et al. (2018) systematically reviewed the influence of air pollution on obesity based on a total of 16 publications [20]. The study identified 66 reported relations between air pollution and body weight. The relationship between air pollutants and body weight with positive, irrelevant, and negative accounted for 44%, 44%, and 12% respectively. In the study of LaKind et al. (2021), 8 studies assessed the association between O_3_ and its effects on weight was included [37]. Only 2 original studies were considered suitable for evidence synthesis and did not find a consistent association between O_3_ exposure and impacts on weight.

Except for the above two systematic reviews, the other 5 meta-analyses studied the relationship between air pollution and obesity, with air pollutants including particular matter (such as PM_1_, PM_2.5_, and PM_10_), NO_2_, SO_2_, and O_3_.

#### Chidren and adolescents

Among the five included meta-analyses, three studies concentrated on the association between air pollutants and obesity among children and adolescents [23, 35, 36]. The studied air pollution included PM_1_, PM_2.5_, PM_10_, nitrogen oxide, and ozone.

***Air pollutant PM***_***1***_ The relationship between air pollutant PM_1_ and the risk of obesity was studied in one meta-analysis [23]. Based on the meta-analysis consisting of two cross-sectional studies with a total of 54,615 cases, PM_1_ significantly raised the risk of obesity among children and adolescents (OR = 1.41, 95% CI: 1.30 ~ 1.53).

***Air pollutant PM***_***2.5***_ This umbrella review included three meta-analyses concentrated on air pollutant PM_2.5_ [23, 35, 36]. Total evidence indicated a positive correlation between PM_2.5_ and obesity. A meta-analysis found an association between PM_2.5_ exposure and obesity in children and adolescents (OR = 1.28, 95% CI: 1.13 ~ 1.45) [23]. In addition, on the basis of a meta-analysis consisting of 6 primary studies, evidence was also found to support that PM_2.5_ exposure could significantly increase the risk of childhood obesity (OR = 1.06, 95% CI: 1.02 ~ 1.10) [35]. However, for body weight status, there was no significant correlation between PM_2.5_ exposure and BMI (β = 0.04, 95% CI: -0. 10 ~ 0. 17) [36].

***Air pollutant PM***_***10***_ There are three meta-analyses that evaluated the association between PM_10_ exposure and the risk of obesity [23, 35, 36]. In detail, a meta-analysis by merging results from 15 studies indicated that PM_10_ was correlated with obesity (OR = 1.12, 95% CI: 1.06 ~ 1.18) [23]. Another study investigated the influence of air pollutants on childhood obesity and found that PM_10_ was one of the factors for the increased risk of obesity (OR = 1.07, 95% CI: 1.04 ~ 1.10) [35]. Also, PM_10_ exposure was significantly related to increased BMI (*r* = 0.034, 95% CI: 0.007 ~ 0.061) based on the pooled analysis of 5 studies [36].

***Air pollutant NO***_***2***_ A total of two meta-analyses evaluated the effect of nitrogen oxide on obesity. Total evidence indicated a significant positive relationship between NO_2_ and increased risk of obesity, and the pooled OR (95% CI) were 1.11 (1.06,1.18) and 1.10 (1.04, 1.16), respectively [23, 35].

***Air pollutant O***_***3***_ Only one meta-analysis studied the relationship between O_3_ and the increased risk of obesity and no significant correlation was found (OR = 1.08, 95% CI: 0.99 ~ 1.18) [23].

#### Adults

Of the five included meta-analyses, only one study focused on the relationship between air pollution and obesity among adults [21]. The studied air pollution included PM_2.5_, PM_10_, NO_2_, SO_2_, and O_3_. For PM_2.5_ exposure, no significant correlation was observed with obesity (OR = 1.21, 95% CI: 0.94 ~ 1.56), while a significant relation was found with BMI (β = 0.34, 95% CI: 0.30 ~ 0.38). The study also found a positive correlation between increased risk of obesity and PM_10_ (OR = 1.06, 95% CI: 0.98 ~ 1.14), NO_2_ (OR = 1.13, 95% CI: 1.01 ~ 1.26), SO_2_ (OR = 1.04, 95% CI: 1.01 ~ 1.06), and O_3_ (OR = 1.07, 95% CI: 1.02 ~ 1.13).

#### The mixed population

One meta-analysis out of five studied the effect of air pollution on obesity and the pollutants considered including PM_2.5_ and PM_10_ among the mixed population of children, adolescents, and adults [22]. The study found that PM_2.5_ could increase the risk of obesity (RR: 1.159, 95% CI: 1.111–1.209) per 10 µg/m^3^ increment, and 5 other articles with maternal exposure showed that PM_2.5_ increased the risk of obesity in children (RR: 1.06, 95% CI: 1.02–1.11). Besides, exposure to particular matter PM_10_ could increase the risk of obesity (RR: 1.092, 95% CI: 1.070–1.116) per 10 µg/m^3^ increment.

## Discussion

This research identified 2 systematic reviews and 5 meta-analyses. Overall, positive relationships between air pollutants (PM_1_, PM_2.5_, PM_10_, NO_2_, SO_2_, and O_3_) exposure and weight status (obesity, overweight, BMI) were found in most studies. At the same time, no significant correlations were also observed in some studies. The influence of air pollution on obesity varied by different ambient air pollutants.

The finding in this study indicated that the risk of obesity had significantly positive associations with PM_1_, PM_2.5_, PM_10_, and NO_2_ among children and adolescents, while there was no significant correlation with O_3_. In addition, systematic reviews and meta-analyses on SO_2_ and obesity still need to be further explored. Previous research showed that higher SO_2_ in childhood was associated with lower BMI among adolescents [38]. While another study indicated that SO_2_ could significantly increase zBMI [39]. The relevant studies are still limited, which can be carried out more in the future. For adults, except for PM_2.5_, PM_10_, NO_2_, SO_2_, and O_3_ were shown significantly positive associations with obesity. However, the relationship between obesity and PM_1_ is still unknown. Studies have reported that exposure to PM_1_ was statistically related to the increased risk of obesity among adults [11, 40].

As we know, obesity is closely related to the occurrence of many chronic diseases such as hypertension, cardiovascular diseases, diabetes, and tumors, and has become one of the major risk factors threatening human health [41–44]. The underlying biological mechanism by which air pollutants contributed to the development and progression of adverse outcomes in body weight remains unclear. Considering that obesity is a complex phenotype, environmental, metabolic, genetic, and physical activity may all influence body weight, which makes the etiological association between air pollution and obesity outcome complex and cannot be attributed to a single biological pathway [45, 46]. There are several possible mechanisms linking air pollution to obesity, including biochemical and behavioral pathways.

The effects of air pollutants on body weight can be realized by altering metabolism. Inhalation of air pollutants leads to metabolic disorders in the body and weight loss. Air pollution can affect metabolic function by changing adipose tissue inflammation, oxidative stress, and personal dietary intake [47–49]. Inhaling air pollutants triggers a series of physiological responses that alter immune, inflammatory and respiratory pathways, leading to altered inflammatory biomarkers and increased levels of oxidative stress in the human body [50]. In addition, PM_2.5_ exposure was found to have a negative effect on glucose metabolism, and exposure to polluted environments is detrimental to glucose metabolism [51]. Besides, air pollutants may indirectly influence body weight by enhancing the risk of chronic diseases, including hypertension, cardiovascular disease, and respiratory diseases [52]. Air pollutant exposure has also been linked to reduced lung function, increased blood pressure, and other respiratory symptoms, leading to impaired exercise capacity and performance [53].

Poor air quality can hinder regular physical activity to some extent [20, 54]. For example, when the news about serious outdoor air pollution is reported in the weather forecast, people may be able to reduce the impact of severe air pollution on themselves by reducing outdoor activities [55]. Air pollution can disrupt normal physical activity, and people will pick the choice to reduce their physical activity by sitting or lying down. These behaviors may result in a decrease in net calorie consumption and to some extent significant weight gain, thereby increasing the risk of obesity. As air quality declines, people’s willingness to participate in outdoor activities (e.g., bicycling, walking, and running) also declines [56].

This umbrella review systematically and comprehensively incorporates the information currently available on the relationship between air pollution and the increased risk of obesity outcomes. However, this study has several limitations. Firstly, although comprehensive literature searches were conducted, the number of included systematic reviews and meta-analyses was not very large, with 2 systematic reviews and 5 meta-analyses. Secondly, the quality and strength of the evidence for the selected systematic reviews/ meta-analyses were assessed by AMSTAR 2, the included studies were of low quality. In addition, additional study designs are needed to draw clear conclusions in different contexts since most studies were observational in nature. Thirdly, the increments of air pollutants in the included studies are quite diversified. To provide a pooled quantified result, it is necessary to normalize the effect estimates. Fourthly, the primary studies were based on observational research, making it difficult to understand the mechanisms by which air pollutants affect obesity outcomes. Besides, the estimated effects obtained from the preliminary studies may also be biased since the observational studies were difficult to avoid confounding factors. Even though they controlled for common significant confounders, residual confounders cannot be completely excluded.

This study also provides several suggestions for future reviews. Firstly, systematic reviews and meta-analyses are recommended to be performed according to the standard methodology required by the PRISMA guidelines. Secondly, considering that the findings of this study are based on observational studies, researchers need to assess the risk of bias in the included studies, which are more or less prone to common biases such as observer bias and information bias. Finally, it is also necessary to assess the quality of each included study based on the relevant tools and evaluate the level of evidence for each exposure and outcome pair.

## Conclusion

In conclusion, this umbrella review showed positive associations between air pollution exposure and increased risk of obesity. The influence of air pollution on obesity varied by different ambient air pollutants. These findings further indicate the importance of strengthening air pollution prevention and control. It is also of great importance for future studies to elucidate the possible mechanisms and pathways linking air pollution to obesity.

### Electronic supplementary material

Below is the link to the electronic supplementary material.

Supplementary Material 1

## Data Availability

Data is provided within the manuscript.
