# Supplementary material for: The association between air pollution and obesity: an umbrella review of meta-analyses and systematic reviews
Source: BMC Public Health. 2024 Jul 11;24:1856. doi: 10.1186/s12889-024-19370-4 (PMC11238414; doi:10.1186/s12889-024-19370-4)
Supplement: Supplementary file 1 — Supplementary Material 1 [file 12889_2024_19370_MOESM1_ESM.docx]

Supplementary Table 1. Search strategy until 16 July 2023

|  |  | PubMed | Web of Science | Cochrane | Embase |
| --- | --- | --- | --- | --- | --- |
| #1 | air pollution | 38,895 | 83,773 | 1,119 | 44,757 |
| #2 | air pollutant* | 13,913 | 53,220 | 366 | 17,908 |
| #3 | air quality | 18,269 | 96,512 | 2,231 | 24,181 |
| #4 | fine particle | 2,640 | 72,313 | 324 | 3,939 |
| #5 | particulate matter | 27,944 | 66,143 | 498 | 34,360 |
| #6 | ozone | 26,376 | 73,490 | 1,174 | 34,457 |
| #7 | nitrogen oxide | 1,754 | 54,353 | 299 | 2,276 |
| #8 | sulfur dioxide | 5,474 | 19,832 | 105 | 6,994 |
| #9 | carbon monoxide | 31,980 | 63,192 | 2,634 | 38,704 |
| #10 | #1 OR #2 OR #3 OR #4 OR #5 OR #6 OR #7 OR #8 OR #9 | 124,652 | 452,859 | 7,454 | 156,871 |
| #11 | overweight | 88,606 | 113,691 | 19,731 | 133,086 |
| #12 | obesity | 321,646 | 390,259 | 31,045 | 449,465 |
| #13 | obese | 150,319 | 199,078 | 26,169 | 235,235 |
| #14 | adiposity | 30,737 | 37,689 | 2,759 | 42,085 |
| #15 | body mass index | 237,185 | 287,269 | 42,572 | 339,869 |
| #16 | BMI | 193,976 | 239,170 | 54,528 | 404,721 |
| #17 | weight | 975,066 | 2,209,300 | 119,492 | 1,311,789 |
| #18 | body fat | 37,988 | 156,816 | 19,452 | 50,580 |
| #19 | waist circumference | 34,326 | 45,561 | 10,347 | 53,696 |
| #20 | waist-to-hip | 7,439 | 8,649 | 1,233 | 9,927 |
| #21 | waist-to-height | 2,860 | 3,289 | 233 | 3,720 |
| #22 | visceral fat index | 121 | 6,126 | 1,089 | 174 |
| #23 | fat mass | 26,328 | 92,375 | 13,729 | 37,692 |
| #24 | fat-free mass | 9,399 | 12,104 | 2,327 | 12,409 |
| #25 | #11 OR #12 OR #13 OR #14 OR #15 OR #16 OR #17 OR #18 OR #19 OR #20 OR #21 OR #22 OR #23 OR #24 | 1,428,847 | 2,827,150 | 188,244 | 2,018,594 |
| #26 | #10 AND #25 | 5,722 | 20,914 | 764 | 7,701 |
| #27 | systematic review | 230,422 | 282,575 | 513 | 268,311 |
| #28 | meta-analys* | 312,295 | 220,120 | 4,171 | 222,759 |
| #29 | #27 OR #28 | 416,161 | 391,071 | 4,580 | 375,685 |
| #30 | #26 AND #29 | 91 | 141 | 1 | 96 |
